# Disulfiram and BKM120 in Combination with Chemotherapy Impede Tumor Progression and Delay Tumor Recurrence in Tumor Initiating Cell-Rich TNBC

**DOI:** 10.1038/s41598-018-35619-6

**Published:** 2019-01-18

**Authors:** Ling Wu, Fanyan Meng, Lun Dong, C. James Block, Allison V. Mitchell, Jason Wu, Hyejeong Jang, Wei Chen, Lisa Polin, Qifeng Yang, Q. Ping Dou, Guojun Wu

**Affiliations:** 1 Barbara Ann Karmanos Cancer Institute, Department of Oncology, Wayne State University School of Medicine, 4100 John R, Detroit, MI USA; 2 Comprehensive Cancer Centre of Drum Tower Hospital, Medical School of Nanjing University and Clinical Cancer Institute of Nanjing University, Zhongshan Road, Nanjing, Jiangsu Province P. R. China; 3 Department of Breast Surgery, Qilu Hospital, Shandong University, Jinan, Shandong Province P. R. China; 4 Department of Biology, Purdue University, West Lafayette, IN USA

## Abstract

Tumor initiating cells (TIC) have been suggested as a mechanism for driving chemoresistance and tumor recurrence in human cancers including triple negative breast cancer (TNBC). Significant progress has been made in targeting TICs. However, methods for simultaneously targeting heterogeneous TIC populations are lacking. In this study, we found that treating TNBC cells with chemotherapeutic agents led to a significant accumulation of the ALDH^+^ TIC population. Treating TNBC cells with a disulfiram and copper mixture (DSF/Cu) specifically decreased the ALDH^+^ TIC population and treatment with BKM120, a pan-PI3K inhibitor, significantly decreased the CD44^+^/CD24^−^ TIC population. Furthermore, treatment with DSF/Cu or BKM120 induced higher levels of apoptosis in ALDH^+^ or CD44^+^/CD24^−^ populations, respectively, than in bulk tumor cells. Combining DSF/Cu and BKM120 treatment simultaneously decreased the ALDH^+^ and CD44^+^/CD24^−^ TICs. Using a TNBC tumor xenograft mouse model, we found that DSF/BKM in combination with Taxol significantly reduced the tumor burden and delayed tumor recurrence compared to Taxol treatment alone. Our study is the first of its kind to use two different drugs to abolish two major TIC subtypes simultaneously and inhibit tumor recurrence. These results lay a foundation for developing a novel therapy that can improve chemotherapeutic efficacy.

## Introduction

Triple-negative breast cancer (TNBC) accounts for 15% of all breast cancers with higher percentages in premenopausal African-American and Hispanic women^1–3^. The lack of estrogen receptor (ER) and progesterone receptor (PR) expression, and HER2 overexpression/gene amplification, limits treatment options for TNBC. Chemotherapy remains the major therapeutic option for TNBC treatment. However, while most TNBC patients initially respond to chemotherapy, 30–40% of these patients experience disease relapse. These recurrent tumors are resistant to chemotherapy and eventually progress to metastasis. Novel therapeutic modalities are urgently needed to reduce the tumor recurrence, metastasis and overall mortality associated with chemoresistance in TNBC^4–7^.

Over the past decade, the tumor initiating cell (TIC) hypothesis has been proposed as a mechanism underlying chemo-resistance, tumor recurrence and cancer metastasis^8–11^. Due to slow proliferation and high self-renewal capability, cancer stem cells display significant chemoresistant characteristics and stay dormant in human body for a period of time. Upon stimulation from the tumor microenvironment, TICs are reactivated and generate new tumors. This TIC hypothesis is supported by accumulated experimental evidence in recent years. For example, enhanced aldehyde dehydrogenase (ALDH) activity is a hallmark of cancer stem cells measurable by the aldefluor assay^12,13^. ALDH1A1 and ALDH1A3, two of 19 ALDH isoforms expressed in humans, were generally believed to be responsible for the ALDH activity of TICs^14,15^. ALDH positive (ALDH^+^) subpopulation isolated from cancer cells showed enhanced tumor-initiating capability than non-TIC^12^. Another distinct tumorigenic TIC population is found to be enriched with a CD44^+^/CD24^−^/ESA^+^ phenotype in human breast and other cancers^16,17^. Further studies also showed that isolated CD44^+^/CD24^−^/ESA^+^ cells can self-renew, reconstitute the parental cell line, retain BrdU labeling, and preferentially survive chemotherapy^16^. Given the essential function of stem-like cells in tumorigenesis, chemoresistance and progression, targeting TICs has been recognized as a promising strategy to overcome drug resistance and tumor recurrence. The strategies targeting TICs include targeting TIC related signaling pathways, targeting TIC surface markers, inhibiting ABC transporters, enhancing immune responses, or targeting the TIC microenvironment^18,19^. However, TICs differ in various tumor types and there is not a single biomarker that can be universally exploited to detect and/or isolate TICs from all types of cancer. In addition, the TIC populations isolated from the same tumor may be phenotypically and functionally distinct. Due to the heterogeneous pattern of TICs in tumor, it is unlikely that targeting one TIC subpopulation will be therapeutically sufficient to prevent all TICs’ function. Thus, simultaneously targeting multiple TIC populations or TIC-related signaling pathways is a more viable alternative.

In this study, we investigated the distribution and chemotherapeutic response of the ALDH^+^ and CD44^+^/CD24^−^ TIC subpopulations in a panel of 14 TNBC cell models. We demonstrated the specific inhibitory effect of DSF/Cu on the ALDH^+^, but not the CD44^+^/CD24^−^ cell population in TNBC cells. In addition, we found that the pan-PI3K inhibitor BKM120 specifically targeted the CD44^+^/CD24^−^ subpopulation. By combining DSF/Cu and BKM120, we were able to induce potent apoptosis in both of the ALDH^+^ and CD44^+^/CD24^−^ populations. Moreover, we showed that treatment of DSF/Cu and BKM120 enhanced chemotherapy-mediated killing of bulk TNBC cells *in vitro*. Finally, the combination of DSF and BKM120 with chemotherapeutic agent Taxol showed significantly stronger effect in inhibiting tumor growth and delaying the tumor recurrence than Taxol treatment alone in a TNBC tumor recurrence xenograft mouse model.

## Results

### Two major TIC subpopulations exist in TNBC cells

**Figure 1:**
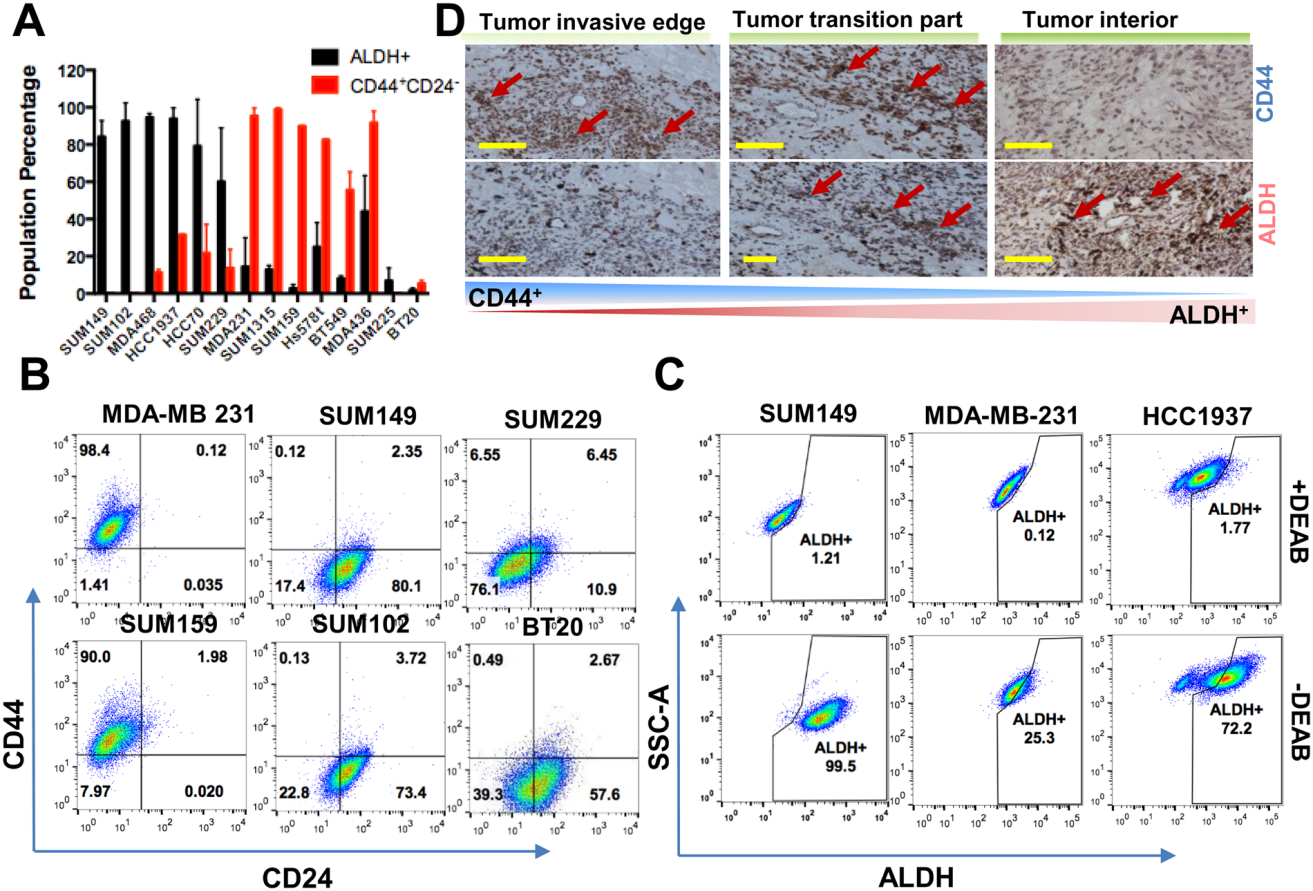
The complex composition of two major TIC populations in TNBC. (**A**) Summary of the composition of the two major TIC subpopulations in a panel of 15 TNBC cell lines. Black: ALDH^+^ population; Red: CD44^+^/CD24^−^ population. Indicated is the mean + SEM of two independent experiments. (**B**) Examples of the CD44^+^/CD24^−^ percentages in six representative TNBC cell models. (**C**) Percentage of the ALDH^+^ subpopulation in a panel of three representative TNBC cell models. DEAB is a specific inhibitor for ALDH1A1. (**D**) Localization of ALDH1^+^, CD44^+^ in the clinical specimen of human invasive breast carcinoma as assessed by immunohistochemistry. Arrows point to positive staining. Bar: 100 μm.

To investigate the distribution pattern of the two major TIC populations, ALDH^+^ and CD44^+^/CD24^−^, in TNBC, we analyzed a panel of 14 TNBC cell lines using fluorescence-activated cell sorting (FACS). We found that within this panel, 42.9% (6/14) exhibited a high proportion (>60%) of ALDH^+^ cells, and 42.9% (6/14) had a high proportion (>80%) of CD44^+^/CD24^−^ cells. 14.2% (2/14) had low proportions (<10%) of both ALDH^+^ and CD44^+^/CD24^−^ cells (Fig. 1A–C). Notably, no cell lines exhibited high proportions of double-positive ALDH^+^ and CD44^+^/CD24^−^ cells. To characterize the TIC populations *in vivo*, we performed immunohistochemical (IHC) analysis on human TNBC tumor specimens. We found that ALDH^+^ cells are mainly localized in the tumor interior, whereas CD44^+^/CD24^−^ cells are enriched at the tumor’s invasive edge. Both populations are present and overlap in the transition region between the invasive edge and interior (Fig. 1D).

### TIC subpopulations in TNBC cells have different characteristics

Although it has long been recognized that ALDH^+^ and CD44^+^/CD24^−^ cells are breast cancer TICs^9,12,20,21^, their oncogenic properties have not been carefully compared within the same cancer cell population. By analyzing the mouse metastatic 4T1 cell model, we identified 5% CD49f^+^/CD24^+^ (the CD44^+^/CD24^−^ counterpart in mouse) and 85% ALDH^+^ cells, as well as a small overlapping fraction representing 2% of the total cell population (Supplementary Fig. 1A). To compare the invasive properties of isolated TICs, we utilized a matrigel invasion assay. Cancer cells expressing either TIC marker were more invasive than bulk tumor cells (Supplementary Fig. 1B). Within TIC-marker-positive cell populations, double-positive cells (ALDH^+^/CD49f^+^/CD24^+^) exhibited both the greatest invasive and self-renewal capacities (Supplementary Fig. 1B). Cells with either ALDH^+^ or CD49f^+^/CD24^+^ alone were comparable in tumorsphere formation capacity, however, displayed diminished sphere formation relative to the double-marker positive, ALDH^+^/CD49f^+^/CD24^+^, TIC population (Supplementary Fig. 1C). This data is consistent with several published papers indicating that ALDH^+^/CD44^+^/CD24^−^ population in fact has the greatest tumorigenic and metastatic capacity^22–24^. Moreover, we found that ALDH^+^ cells had a typical epithelial morphology, while CD49f^+^/CD24^+^ cells exhibited a mesenchymal morphology (Supplementary Fig. 1D). This finding was confirmed by immunostaining, which showed positive staining of the epithelial markers β-catenin and E-cadherin in ALDH^+^ cells and mesenchymal markers vimentin and fibronectin in the CD49f^+^/CD24^+^ population (data not shown). Finally, we used a repopulation assay to compare the self-renewal capabilities of the TIC populations. We found that after being cultured for 20 days, the ALDH^+^ cells demonstrated a strong capability to generate an ALDH^+^ population, and the CD49f^+^/CD24^+^ population had a strong capability to generate a CD49f^+^/CD24^+^ population. The overlapping population demonstrated a capability to generate both TIC populations (Supplementary Fig. 1E).

### Chemotherapeutic agents enriched TIC populations in the residual cancer cells

**Figure 2:**
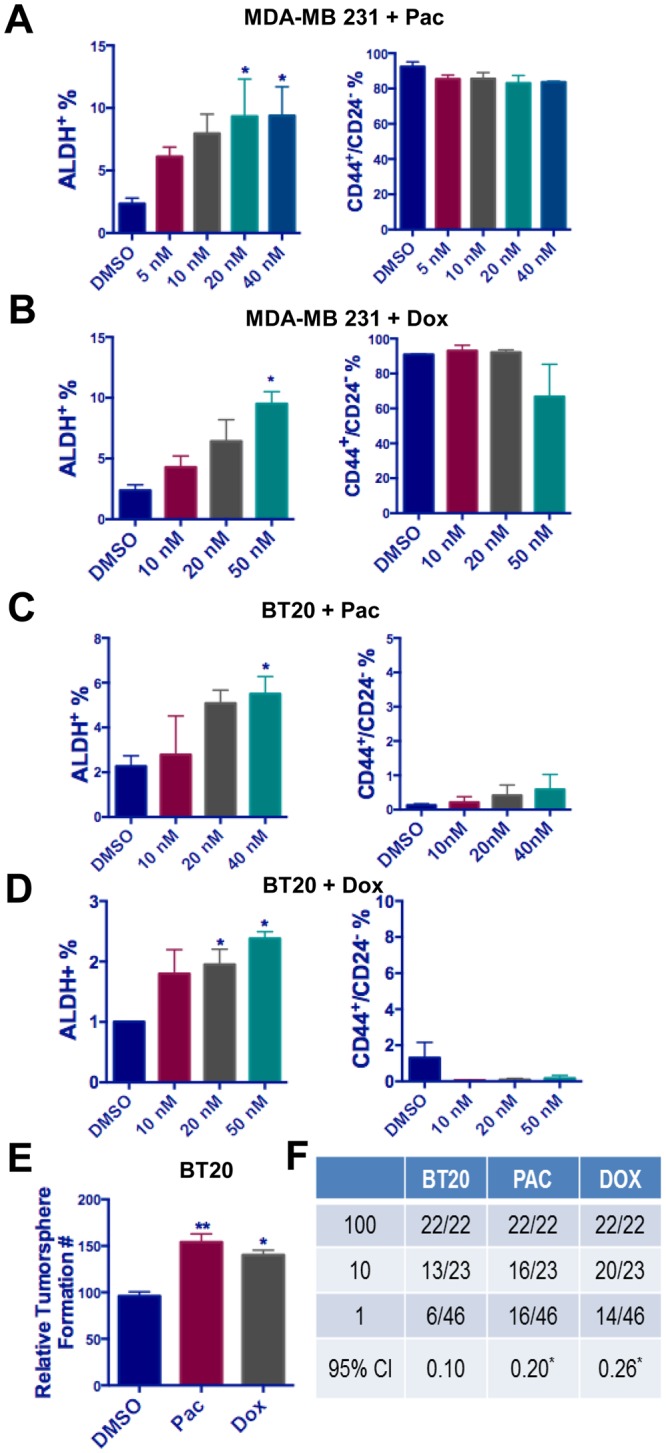
The treatment of paclitaxel and doxorubicin leads to enrichment of ALDH+ TIC in TNBC. (**A**,**B**) The MDA-MB231 cells were treated with various doses of paclitaxel (**A**) and doxorubicin (**B**) for 72 hours. The ALDH^+^ and CD44^+^/CD24^−^ subpopulation was examined using flow cytometry. (**C**,**D**) The BT20 cells were treated with various doses of paclitaxel (**C**) and doxorubicin (**D**) for 72 hours. The ALDH^+^ and CD44^+^/CD24^−^ subpopulation was examined using flow cytometry. (**E**) The BT20 cells showed increased self- renewal capability after chemotherapy (40 nM Pac or 50 nM Dox) for 72 hours as measured by tumorsphere formation assay. For panel (A–E), one-way ANOVA followed by multiple comparisons was used with *P* < 0.05 as statistically significant. (**F**) Results of limiting dilution analysis for secondary sphere formation of BT20 cells with and without Pac or Dox treatment. Data were analyzed using the ELDA software. *Indicates significant difference from untreated group by Pac or Dox. Pr = 0.0164 for Pac treated group and 0.000796 for Dox treated group.

Chemotherapy is the main treatment option for TNBC patients, for whom targeted therapies are unavailable. Although most TNBC cases initially respond to chemotherapy, 30–40% eventually develop recurrent disease^4,25,26^. We hypothesized the TICs are one of the main contributors to TNBC recurrence. To test this hypothesis, we examined whether treatment with one of two first-line chemotherapeutic agents, paclitaxel (Pac) or doxorubicin (Dox), could enrich TIC populations *in vitro*. We first examined the effect of treatment in MDA-MB231 cells, which exhibits a high CD44^+^/CD24^−^ and low ALDH^+^ population. Treatment with either Pac or Dox for 72 hours led to a significant, dose-dependent increase in the ALDH^+^ cell fraction in MDA-MB231, without inducing a marked change in the CD44^+^/CD24^−^ population (Fig. 2A,B). We next examined this effect in BT20 cells, which have low proportions of both stem cell populations. Again, treatment with either Pac or Dox for 72 hours led to a highly significant increase in the ALDH^+^ fraction and a relatively minor increase in the CD44^+^/CD24^−^ (Fig. 2C,D). Next, we generated Pac-resistant SUM159 cells (SUM159-PacR) by gradually increasing Pac concentration and observed dose-dependent increase of ALDH^+^ population without changes in CD44^+^/CD24^−^ population (Supplementary Fig. 2). Functionally, we observed a consistent increase in tumorsphere formation in BT20 cells after treatment with Pac or Dox (Fig. 2E). The effect of Pac and Dox treatment on BT20 cells self-renewal capability was further validated using a sphere limiting dilution assay (Fig. 2F). These data indicate that enrichment of the ALDH^+^ population is a common response of TNBC cells to various chemotherapeutic agents.

### DSF/Cu treatment led to selective decrease in the ALDH^+^ cell population

**Figure 3:**
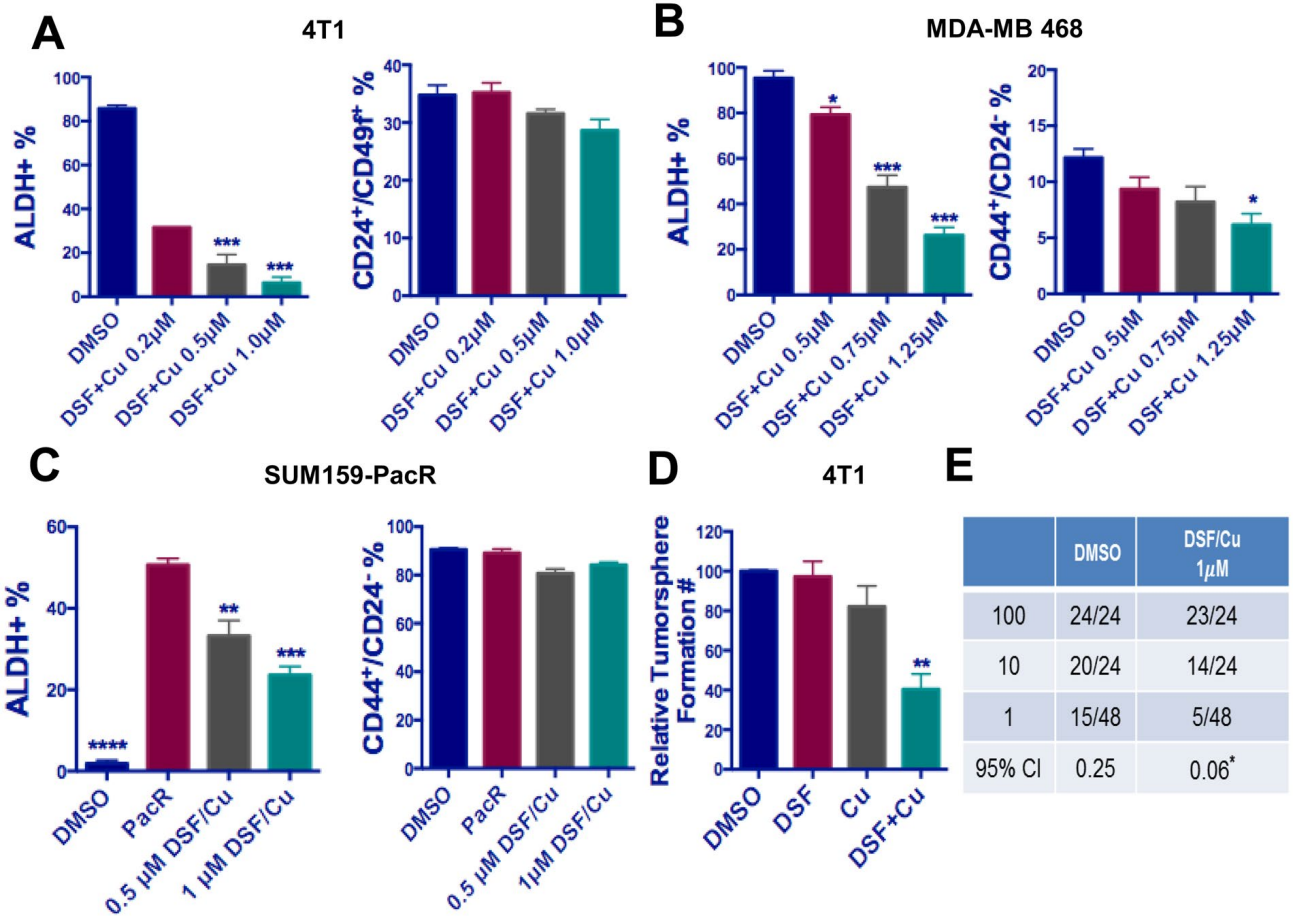
The effect of DSF on the ALDH^+^ TIC population. (**A**) DSF/Cu significantly decreased the ALDH^+^ but not the CD44^+^/CD24^−^ TIC population in 4T1 cells in a dose dependent manner. (**B**) DSF/Cu significantly decreased the ALDH^+^ but not the CD44^+^/CD24^−^ TIC population in MDA-MB468 cells in a dose dependent manner. (**C**) DSF/Cu decreased the ALDH^+^ in a dose-dependent manner, but not the CD44^+^/CD24^−^ TIC population in Pac-resistant SUM159 cells. (**D**) DSF/Cu mixture, but not DSF or Cu^2+^ alone, significantly resulted in significantly decreased tumorsphere formation in 4T1 cells. For panel (A–D), one-way ANOVA followed by multiple comparisons was used with *P* < 0.05 as statistically significant. (**E**) Results of limiting dilution analysis for secondary sphere formation of 4T1 cells with and without DSF/Cu treatment. Data were analyzed using the ELDA software. *Indicates significant difference from untreated group by DSF/Cu. Pr = 2.4 × 10^−6^.

The promising results obtained from repurposing DSF/Cu for cancer therapy^27–29^ encouraged us to investigate its effect on TICs^30–33^. We expanded our scope by including human TNBC cell models SUM102 and MDA-MB468, which contain a high proportion of ALDH^+^ TIC population like 4T1 cells. Similar to the effect on bulk tumor cells, individual treatment with DSF or Cu alone had no significant effect on the ALDH^+^ population in 4T1 and SUM102 cells. However, treatment with a DSF/Cu mixture significantly reduced the ALDH^+^, but not the CD44^+^/CD24^−^ population in both 4T1 and SUM102 cells (Supplementary Fig. 3A–D). More importantly, treatment with a DSF/Cu mixture on 4T1 and MDA-MB468 cells reduced ALDH^+^ population in a dose-dependent manner, with no significant change in the CD44^+^/CD24^−^ cell population (Fig. 3A,B). This result was confirmed in SUM102 cell lines, which showed at least a 45% decrease in the ALDH^+^ population (Supplementary Fig. 4A,B). Additionally, treatment of DSF/Cu also decreased ALDH^+^ in SUM159-Pac cells (Pac-resistant) in a dose-dependent manner (Fig. 3C). However, treatment with either Pac or DSF/Cu caused little change in the CD44^+^/CD24^−^ population in SUM159-Pac cells (Fig. 3C). Functionally, we observed that DSF/Cu treatment significantly decreased the self-renewal capability by a tumorsphere formation assay and sphere limited dilution assay in 4T1 cells (Fig. 3D,E). These results indicate that DSF/Cu treatment could be a selective and effective option for targeting ALDH^+^ TICs in TNBC cells.

### BKM120, a clinically used pan-PI3K inhibitor, significantly decreased the CD44^+^/CD24^−^ TIC population in TNBC

**Figure 4:**
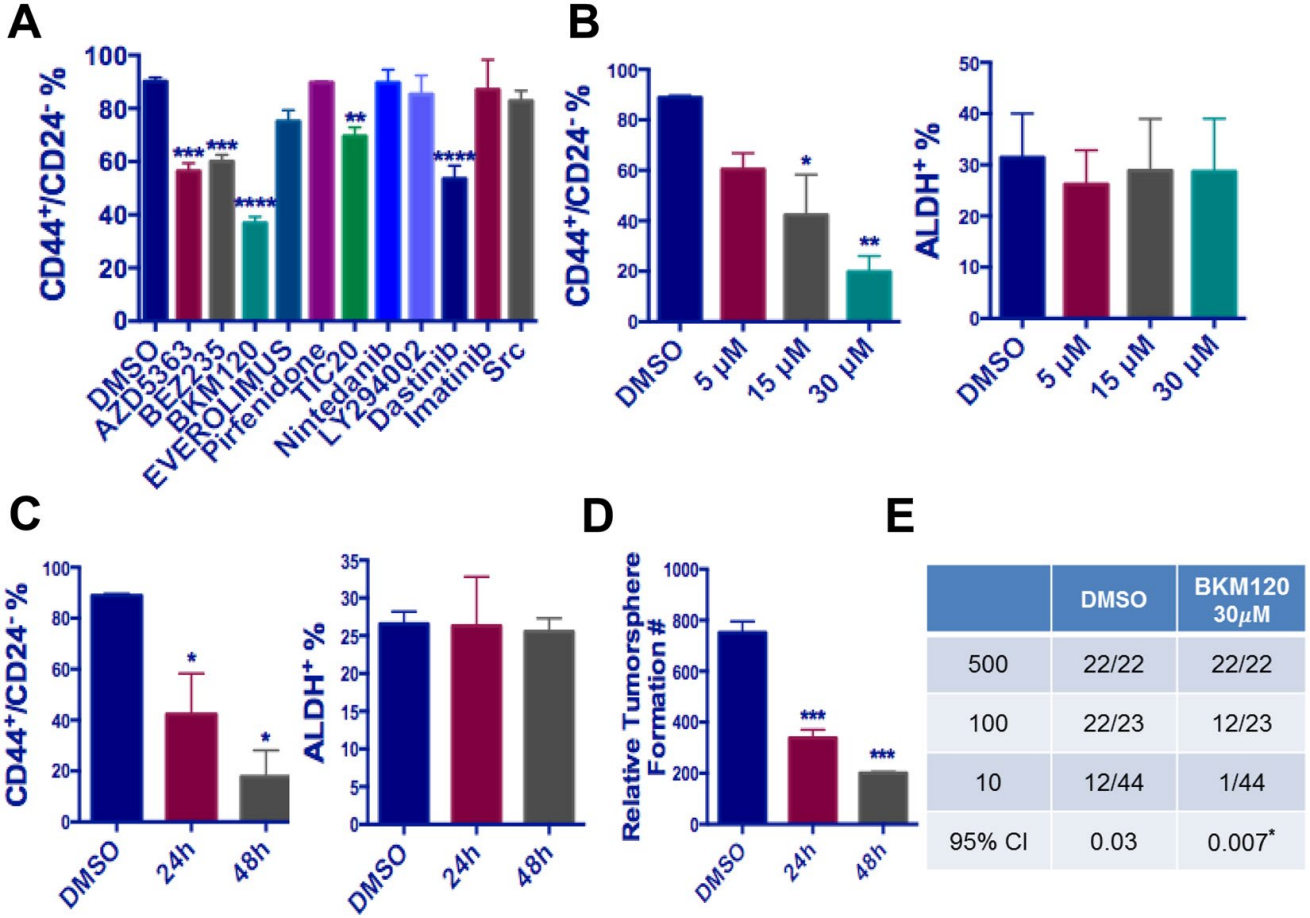
Identification of BKM120 as a CD44^+^/CD24^−^ TIC-targeting drug. (**A**) Screen of a panel of clinically used drugs identified BKM120, a pan- PI3K inhibitor, as a potent CD44^+^/CD24^−^ cell targeting drug in MDA-MB231 cells. (**B**) BKM120 showed dose-dependent effects in decreasing CD44^+^/CD24^−^ (left panel) but not ALDH^+^ (right panel) TIC populations in MDA-MB231 cells. MDA-MB231 cells reated with 5 µM, 15 µM and 30 µM BKM120 for 24 hours were analyzed for TIC population with FACS. (**C**) BKM120 showed time-dependent effects in decreasing CD44^+^/CD24^−^ (left panel) but not ALDH^+^ (right panel) TIC populations in SUM102 cells. SUM102 cells were treated with 15 μM BKM120 for 24 hour or 48 hours were analyzed for TIC population with FACS. (**D**) BKM120 showed a time-dependent effect in inhibiting tumorsphere formation in MDA-MB231 cells. For panel (A–D), one-way ANOVA followed by multiple comparisons was used with *P* < 0.05 as statistically significant. (**E**) Results of limiting dilution analysis for secondary sphere formation of MDA-MB231 cells with and without BKM120 treatment. Data were analyzed using the ELDA software. *Indicates significant difference from untreated group by BKM120. Pr = 2.14 × 10^−6^.

While DSF/Cu showed promising effects on selectively targeting the ALDH^+^ population, it did not significantly inhibit the CD44^+^/CD24^−^ population. These findings prompted us to identify a drug that could selectively target the CD44^+^/CD24^−^ TIC population and could be combined with DSF/Cu to eradicate both major TIC subpopulations simultaneously. To do so, we performed a small-scale drug screen using MDA-MB231 cell line, which contains a high proportion of CD44^+^/CD24^−^ TICs. Nine agents, including several anticancer drugs being tested in clinical trials, were used in this screening (Fig. 4A). Among these drugs, we preferentially selected several drugs that target AKT signaling components because the AKT signaling has been reported to be associated with the CD44^+^/CD24^−^ population^34–36^.

Our data showed that with a near-IC_50_ dose, AZD5363 (pan-AKT inhibitor)^37,38^, BEZ235 (dual ATP-competitive PI3K and mTOR inhibitor)^39,40^, and BKM120 (PI3K inhibitor)^41,42^ reduced the CD44^+^/CD24^−^ population by more than 20%. BKM120 showed the highest reduction of 40% (Fig. 4A). After MDA-MB231 cells were treated for 24 h with BKM120, there was a strong dose-dependent inhibition of the CD44^+^/CD24^−^ population with limited effects on the ALDH^+^ population (Fig. 4B). We also observed a time-dependent effect of BKM120 (at 15 µM) in inhibiting the CD44^+^/CD24^−^, but not ALDH^+^ TIC population in MDA-MB231 cells (Fig. 4C). Similar results were observed in SUM159 cells after BKM120 treatment (data not shown). Consistent with the decrease in the CD44^+^/CD24^−^ population, BKM120 treatment significantly inhibited self-renewal capability of MDA-MB231 cells as measured by a tumorsphere formation assay and a sphere limiting dilution assay (Fig. 4D,E).

### DSF/Cu and BKM120 induced apoptosis in TIC populations

**Figure 5:**
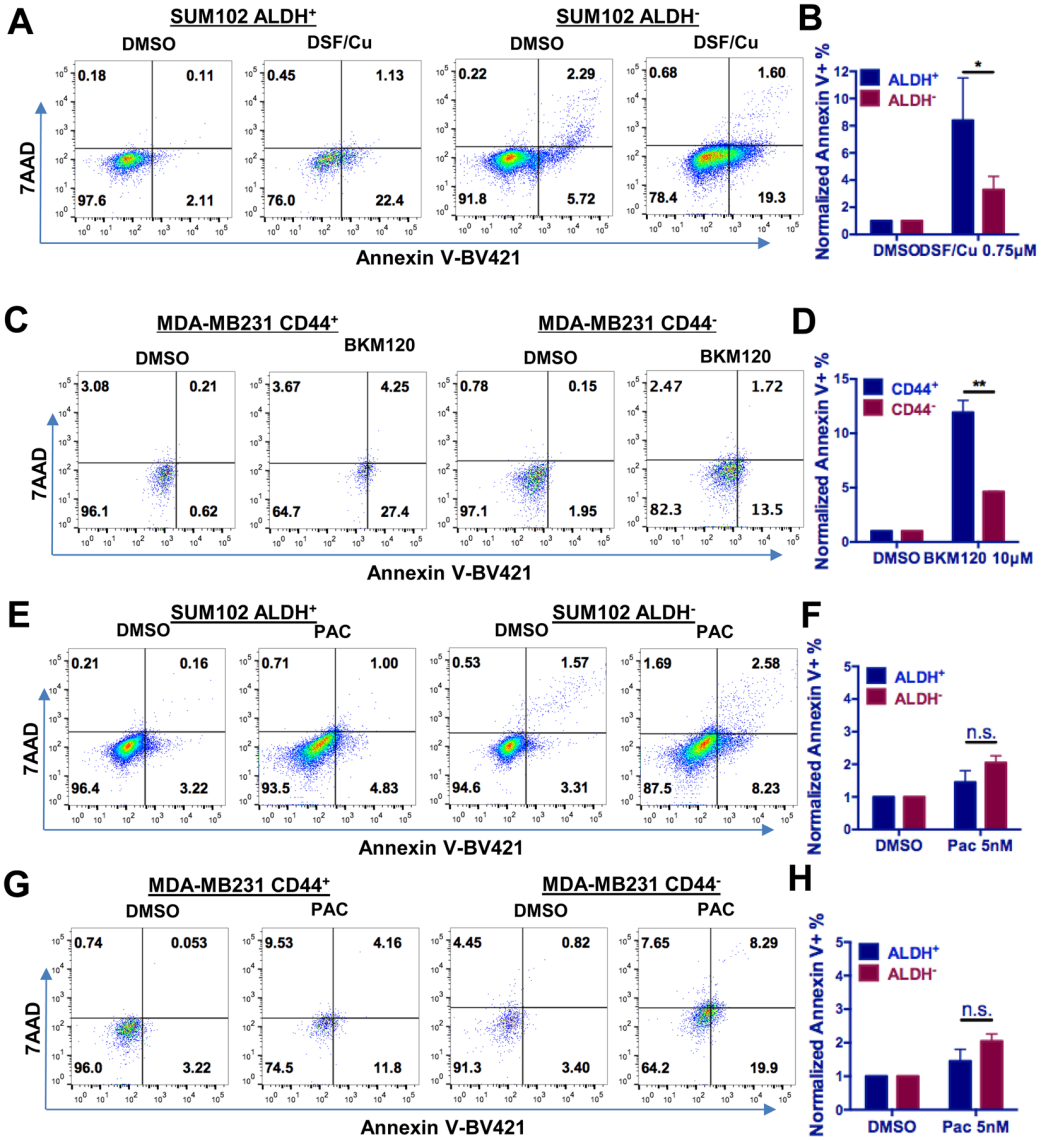
DSF/Cu and BKM120 induce apoptosis in corresponding TIC populations. (**A**) The FACS analysis of apoptotic cells with or without DSF/Cu mixture treatment (0.75 µM) in ALDH^+^ and ALDH^-^ population in SUM102 cells. (**B**) DSF/Cu mixture treatment in SUM102 cells led to more profound apoptosis in the ALDH^+^ population compared to the ALDH- population (*P < 0.05). (**C**) The FACS analysis of apoptotic cells with or without BKM120 treatment (10 µM) in CD44^+^ and CD44^−^ population in MDA-MB231 cells. (**D**) BKM120 treatment (10 µM) in MDA-MB231 cells led to more profound apoptosis in the CD44^+^ TIC population compared to the CD44^−^ population (**P < 0.01). (**E**) The FACS analysis of apoptotic cells with or without PAC treatment in ALDH^+^ and ALDH^−^ population in SUM102 cells. (**F**) Summary of the FACS assay results showed no significant change of apoptosis between ALDH^+^ and ALDH^−^ cell population after PAC treatment. (**G**) The FACS analysis of apoptotic cells with or without PAC treatment in CD44^+^ and CD44^−^ population in MDA-MB231 cells. (**H**) Summary of the FACS assay results showed no significant change of apoptosis between CD44^+^ and CD44^−^ cell populations after PAC treatment.

Since we found that DSF/Cu and BKM120, respectively, targeted the ALDH^+^ or CD44^+^/CD24^−^ TIC populations, we hypothesized that these drugs might selectively induce apoptosis in each TIC population. SUM102 cells, which exhibit a high ALDH^+^ and a low CD44^+^/CD24^−^ subpopulation, were treated with DSF/Cu for 24 hours, and the percentage of annexin V-positive apoptotic cells was determined for both the ALDH^+^ and ALDH^−^ subpopulations. DSF/Cu (at 1 µmol/L) induced a higher level of apoptosis in the ALDH^+^ cells than in the ALDH^−^ cells (Fig. 5A,B). Similarly, BKM120 caused more pronounced apoptosis in the CD44^+^/CD24^−^ cell population than in non-CD44^+^/CD24^−^ cell population in the MDA-MB231 cell line, which has a high CD44^+^/CD24^−^ and a low ALDH^+^ subpopulation (Fig. 5C,D). We next specifically investigated the apoptosis pattern in both cell lines after Pac treatment. We found that Pac treatment induced a similar level of apoptosis in non-TICs compared with TICs in both cell models (Fig. 5E–H).

The above results were validated with an immunofluorescence assay. For SUM102 cells treated with DSF/Cu for 24 hours, apoptosis was assessed by double staining with antibodies to ALDH1A1 and p27. DSF/Cu induced much stronger p27 staining in the ALDH^+^ cell population than ALDH^−^ cell population (Supplementary Fig. 5A). Similarly, BKM120 induced much stronger p27 staining in the CD44^+^ cell population than in the CD44^−^ cell population in MDA-MB231 cells (Supplementary Fig. 5B).

### DSF/Cu and BKM120 decreased the total TIC and sensitized chemotherapy in TNBC cells

**Figure 6:**
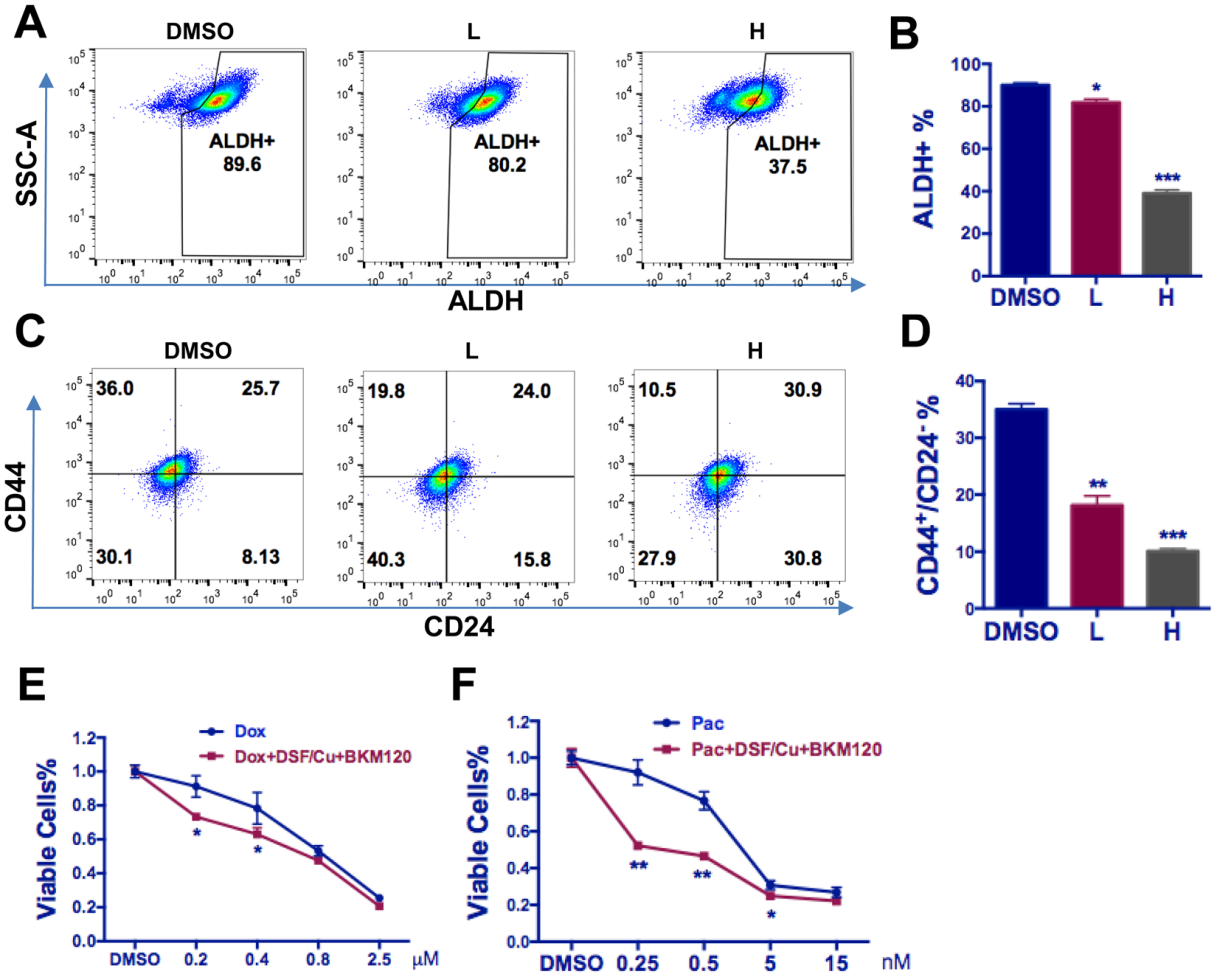
Combinational effect of DSF/Cu and BKM120 on TIC population and bulk cancer cells. (**A**,**C**) Representative figures show dose dependent decrease of ALDH^+^ (**A**) and CD44^+^/CD24^−^ (**C**) population due to the co-treatment of DSF/Cu and BKM120. L: Low dose (DSF/Cu 0.1 µM and BKM120 1 µM). H: High dose (DSF/Cu 0.5 µM and BKM120 5 µM). (**B**,**D**) Summary of the co-treatment effect of DSF/Cu and BKM120 mixture on TIC populations. L: Low dose (DSF/Cu 0.1 µM and BKM120 1 µM). H: High dose (DSF/Cu 0.5 µM and BKM120 5 µM). For panel B-D, one-way ANOVA followed by multiple comparisons was used with *P* < 0.05 as statistically significant. (**E**,**F**) Low dose of DSF/Cu and BKM120 mixture (0.075 µM and 1.125 µM, respectively) sensitizes MDA-MB468 cells to chemotherapeutic agent Dox (**E**) and PAC (**F**) DSF/Cu and BKM120 mixture along with indicated chemotherapeutic agents were added to MDA-MB468 cells for 24 hours and then were replaced with normal medium for another 24 hours before measured by MTT. For panel E-F, two-way ANOVA with multiple comparison was used with *P* < 0.05 as statistically significant.

We next investigated the combined effect of DSF/Cu and BKM120 on the total TIC population in breast cancer cells. Different doses of DSF/Cu and BKM120 [high doses (0.5 and 5 µM), and low doses (0.1 and 1 µM)] were used to treat HCC1937 cells, a cell model exhibiting roughly equal expression of both TIC subpopulations. As expected, the ALDH^+^ and CD44^+^/CD24^−^ cell populations were simultaneously decreased with the higher doses of DSF/Cu and BKM120 indicating that DSF/Cu and BKM120 together decreased the total TIC population in TNBC cells (Fig. 6A–D).

Next, we explored the potential chemosensitizing effect of DSF/Cu combined with BKM120. We found that combined low doses of DSF/Cu (0.075 µM) and BKM120 (1.125 µM) could sensitize MDA-MB468 cells to Dox (0.2 and 0.4 µM, *P < 0.05) (Fig. 6E) and Pac (0.25 to 5 nM, **P < 0.01) (Fig. 6F). These results suggest combining DSF/Cu and BKM120 with chemotherapy could decrease the necessary dose of chemotherapeutic drugs and thus reduce the potential toxicity related to chemotherapy.

### DSF and BKM120 cooperate with chemotherapy to inhibit tumorigenesis and delay tumor recurrence *in vivo*

To investigate DSF and BKM120 effect *in vivo*, we performed a pilot study to define the dose range and potential toxicity of these two drugs individually or together in a TNBC cell line MDA-MB468 tumor xenograft model. Six different treatment schedules were performed with different drug dosages for a duration of 15 days (Supplementary Table 1). We found two different dose combinations of DSF and BKM120 showed an active effect on tumor regression (%T/C 22 (high dose) vs 43 (low dose)). Additionally, most treatments were well tolerated and effective at inhibiting MDA-MB 468 tumor growth, except for one mouse fatality in the high dose combination group, which resulted in adjustment of the maximum dose of BKM120.

**Figure 7:**
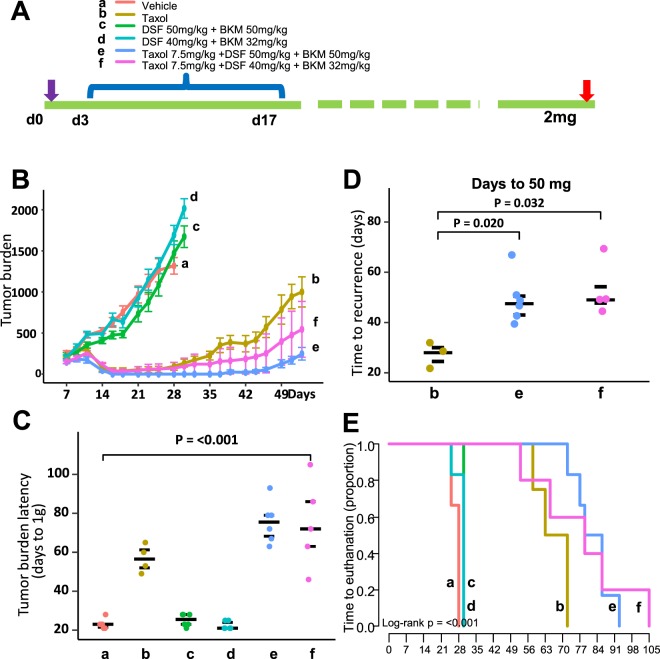
The effect of combination therapy. (**A**) The therapeutic schedule to examine the effect of DSF-BKM120 mixture in combination with chemotherapy for TNBC treatment. There are six different treatment groups: (a) Vehicle; (b) Taxol; (c) 50 mg/kg DSF and 50 mg/kg BKM; (d) 40 mg/kg DSF plus 32 mg/kg BKM; (e) 7.5 mg/kg Taxol plus 50 mg/kg DSF and 50 mg/kg BKM; and (f) 7.5 mg/kg Taxol plus 40 mg/kg DSF and 32 mg/kg BKM. (**B**) The effect of 6 different treatments on overall tumor burden (mg) before first mouse in treatment group reached ending point. (**C**) The effect of 6 different treatments on tumor burden latency (days to reach end points). (**D**) The effect of treatments including Taxol alone (b group), high (e group) and low (f group) dose of DSF-BKM120 mixture in combination with Taxol on tumor recurrence. (**E**) The Kaplan-Meier survival plot showed the time of mice in 6 treatment groups reached ending points. Only the high dose DSF-BKM120 mixture in combination with chemotherapy demonstrated statistical significance in comparison with Taxol treatment alone (*P* < 0.001).

We next evaluated *in vivo* effect of Taxol, BKM120 and Disulfiram in combination against the TNBC tumor xenograft model MDA-MB468. Mice were randomly assigned into six groups and treatment initiated on day 3 post implant (early stage disease). Detailed information regarding the dose and combination, as well as the treatment schedule is shown in Fig. 7A and Supplementary Table 2. Overall, no adverse toxicity was observed in any of the treatment groups other than transient weight loss (Group b: Taxol − weight loss nadir = 1.6% on day 4; full recovery day 6) and Group e: DSF+ BK + Taxol high dose combination: 3.6% weight loss nadir on day 4; full recovery day 7). The combined treatment regimens of DSF plus BKM120 was less active (Groups c and d produced a 66% and 93% T/C respectively on day 21 (2 days post last treatment) compared to Taxol and other two Taxol-including combinations. We found that Taxol treatment alone significantly inhibited tumor growth (0% T/C on day 21) with a median tumor growth delay (T-C) of 34.5 days (Fig. 7B). More importantly, both the high and low dose combination therapies (Groups e and f: DSF/BKM120/taxol) significantly extended tumor burden latency (time to 1 g) to 79 or 75 days vs 58 days for Taxol chemotherapy alone (Fig. 7C), and additionally, significantly delayed tumor recurrence (53 and 49.5 days respectively) to a greater extent than Taxol given as single agent therapy (27 days) (Fig. 7D). Finally, the high dose (group e), but not the low dose (group f), combination therapies demonstrated statistical significance in improving the time to euthanasia compared to Taxol treatment alone (group b) (*P* < 0.001) (Fig. 7E), suggesting a potential improvement in mouse survival.

The results were further validated by immunohistochemical evaluation (IHC) of collected tumor samples from different treatments. ALDH1A1 or CD44 staining was significantly decreased in tumors that received the DSF-BKM120 mixture or DSF-BKM120 mixture plus Taxol treatment. Cleaved caspase 3 staining was increased in both the Taxol and DSF-BKM120 combination treated groups, while the combination of these two treatments showed the strongest staining, suggesting apoptosis was induced by different treatments. Using β-tubulin as a differentiation marker, we found that strongest β-tubulin staining was found only in the tumor receiving DSF-BKM120 mixture plus Taxol combined treatment, suggesting increased tumor differentiation (Supplementary Fig. 6).

## Discussion

It is well-recognized that there are TIC subpopulations in cancer harboring self-renewal capabilities very similar to that of embryonic stem cells (ESCs). These TIC subpopulations can regenerate themselves and other cancer cells within an appropriate micro-environmental niche. At this moment, the TIC model is one appropriate model to explain the complex relationship between chemoresistance, tumor recurrence and metastasis. In accordance with this, accumulating evidence from *in vitro* and *in vivo* experimental models, as well as clinical samples, supports the involvement of TICs in chemoresistance, tumor recurrence and metastasis. Importantly, experimental evidence has demonstrated that although in breast cancer both the ALDH^+^ and CD44^+^/CD24^−^ sub-populations have strong self-renewal capability and will regenerate tumor mass when they were implanted in mice, multiple research groups showed that ALDH^+^ and CD44^+^/CD24^−^ populations are not identical in terms of their oncogenic characteristics and cells co-expressing ALDH^+^ and CD44^+^/CD24^−^ may harbor most strongest oncogenic and self-renewal capability^22–24^. This notion was further confirmed most recently by a transcriptional profiling study based on RNA and single cell sequencing^43^. Aligned with these reports, several groups reported that chemotherapy could lead to accumulation of TIC cells *in vitro* and *in vivo*^44–47^. In current study, we showed that only the ALDH^+^ TIC subpopulation was significantly enriched after chemotherapy (Fig. 2). These data suggest that ALDH^+^ TICs may be the major cell subpopulation responsible for recurrence following chemotherapy, further confirming the heterogeneity of TIC population in breast cancer.

Significant progress has been made in identification of effective strategy to target TIC or stem-like cells in different human cancer recent years, with some “TIC-targeted” drugs are moving through the pre-clinical pipeline to clinical stages of development^48–50^. However, very few therapeutic strategies were tested to address the heterogeneity of TIC. Given the facts of the distinct properties of these TIC subpopulations at the molecular and functional levels^23^ and the potential transition between different subpopulations, targeting these TIC populations simultaneously may generate a more complete response and better therapeutic efficacy in terms of inhibiting tumor recurrence, in comparison with chemotherapy alone. This speculation was supported by our results that DSF/Cu and BKM120 in combination with chemotherapy achieved significant delay of tumor recurrence and progression than chemotherapy alone in treating TNBC tumor *in vitro* and *in vivo*. Of note, the inhibitory effect of DSF on ALDH^+^ TIC has been reported^30–33^, but its selectivity and use in combination with a CD44^+^/CD24^−^ TIC targeting agent was first investigated in the current study.

The underlying molecular mechanism of these treatments in our study requires further exploration. Accumulating evidence suggests that PI3K/AKT signaling plays a role in CD44^+^/CD24^−^ TICs^51–54^. Additionally, it has been reported that isoforms of the catalytic subunit of class-IA PI3K have distinct roles in regulating behavior of normal/oncogenic signaling and murine embryonic stem cells^55^. More recently, oncogenic PIK3CA activated multi-potency was found to induce breast tumor heterogeneity^56^. Our study showed that a pan-PI3K inhibitor BKM120 selectively targeted CD44^+^/CD24^−^ over the ALDH^+^ subpopulation (Fig. 4), which is consistent with above published reports. On the other hand, ALDH activity as a cancer stem cell marker has been explored as a DSF/Cu target in several types of human cancers^30–33^, but it remains unclear why DSF/Cu, a specific ALDH inhibitor, can eradicate cancer stemness. One of the possibilities is that DSF/Cu targets certain signaling modules or pathways in stem cells, which are closely related or bound to ALDH activity. Recently, activation of Notch and Wnt signaling pathways have been shown in ALDH^+^ enriched populations isolated from different human cancers including breast, ovarian and liver cancers^57^. A future direction in our research group is to identify and validate the direct therapeutic targets of DSF in ALDH^+^ enriched population of human TNBC cancer cells.

Currently, DSF is being tested in combination with chemotherapy in several clinical trials, and some of the results were not satisfactory^58^. BKM120 has also been used to treat solid tumors either alone or in combination with other chemotherapies^41,42^. Most of the trials are ongoing and the results are unclear. However, most of those studies aimed at killing bulk cancer cells to stop tumor progression and did not consider the co-existence of multiple stem-like cell subpopulations and their possible changes under therapeutic conditions. Our approach specifically addresses the weakness of current therapies and will eliminate two major stem cell populations that harboring distinct characteristics in TNBC. From this point of view, our approach, when in combination with chemotherapy, is expected to improve therapeutic efficacy against tumor progression and recurrence in comparison with chemotherapy alone.

## Materials and Methods

### Cell culture

A total of 8 different human basal-like breast cancer cell lines (MDA-MB231, MDA-MB468, MDA-MB436, BT20, BT549, HCC70, HCC1937, and HS.578t) were obtained from ATCC and cultured as recommended by ATCC. Six SUM series of basal like breast cancer cell lines (SUM149, SUM102, SUM159, SUM1315, SUM225 and SUM229) were provided by Professor Stephen P. Either from the Medical University of South Carolina. SUM159, SUM149 and SUM 225 were cultured in Ham’s F12 medium with 5% FBS with supplementary of 5 µg/ml insulin and 1 µg/ml hydrocortisone (5% IH), SUM 1315 cells were cultured in F12 medium with 5% FBS with supplementary of 5 µg/ml insulin and 10 ng/ml EGF (5% IE). SUM102 cells were cultured in F-12 medium with supplementary of serum replacement factors (5 mM ethanolamine, 10 mM HEPEs, 5 µg/ml transferrin, 10 nM triiodothyronine, 50 µM sodium selenite, and 0.5 g/L BSA), 5 µg/ml insulin, 1 µg/ml hydrocortisone and 10 ng/ml EGF (SFIHE). The mouse breast cancer cell line 4T1 was obtained from ATCC and was cultured in high glucose DMEM and supplemented by 10% FBS, NEAA and antibiotics (100 U/ml penicillin and 100 μg/ml streptomycin).

### Flow Cytometry analysis

Cells were harvested with trypsin treatment and washed with PBS containing 2% FBS and 2% BSA. The ALDEFLUOR kit (Stem Cell Technologies, Durham, NC, USA) was used to isolate the population with a high ALDH enzymatic activity. Cells were suspended in ALDEFLUOR assay buffer containing ALDH substrate (BAAA, 1 mmol/L per 1 × 10^6^ cells) and incubated for 20 mins at 37 °C. As negative control, for each sample of cells an aliquot was treated with 50 mmol/L diethylaminobenzaldehyde (DEAB), a specific ALDH inhibitor. Samples were then analyzed using a BD LSR II flow cytometer (BD Biosciences). For CD44/CD24 labelling, combinations of fluorochrome-conjugated monoclonal antibodies obtained from BD Biosciences (San Diego, CA, USA) against human CD44 (FITC; cat. #555478) and CD24 (PE; cat. #555428) or their respective isotype controls were added to the cell suspension at concentrations recommended by the manufacturer and incubated at 4 °C in the dark for 30 mins. The labeled cells were washed in the wash buffer, and then analyzed on a BD LSR II flow cytometer (BD Biosciences). At least 20, 000 cells were counted. To analyze stem cell repopulation capability, the unsorted 4T1 cells and the sorted CD49f^+^/CD24^+^ and ALDH^+^ sub-populations (1 × 10^4^) were placed in regular medium for 10 days, and the resulting cell populations were reanalyzed by FACS for ALDH, CD24, and CD49f.

### Cell proliferation assay

Cells were seeded in triplicate at the density of 2.5 × 10^3^ per well in 96-well plates on day 0. The cells were treated with chemotherapy agents Paclitaxel (Pac) and Doxorubicin (Dox) or in combination with DSF/Cu and BKM120 for 24 hours with different doses as indicated. After being cultured in a drug-free growth medium for another 24 hours, the surviving cells were quantified by MTT assay (Life technology). All these experiments were repeated two times.

### Cell migration and invasion analysis

Cell migration and invasion assays were done as previously described^59^. Briefly, Cell migration and invasion assay was performed using the 24-well control chamber and Matrigel invasion chamber, respectively, according to the manufacturer’s instructions (BD Biosciences, San Jose, CA). All cell lines were seeded in a density of 1.0 × 10^4^ /chamber with full culture medium without FBS. Medium with 10% FBS was used as a chemoattractant. 24 hours after seed, migratory and invading cells were fixed and stained with Diff-Quik kit.

### Apoptosis detection

Cells were seeded at 1 × 10^5^ per ml on 25 cm^2^ flask overnight before treated with different drugs at various concentrations for 24 hours. Determination of apoptotic cells by fluorescent staining was done as described previously^59^. Briefly, cells were incubated with BV421-annexin V and 7AAD (BD Biosciences) in binding buffer for 15 minutes in dark. Stained cells were immediately subjected to flow cytometry analyses using BD LSR II flow cytometer (BD Biosciences). Moreover, apoptotic cells could be detected with Immunostaining with Apoptotic markers p27. The images were taken with Nikon inverted Microscope Eclipse Ti-E system.

### Tumorsphere formation and sphere limiting dilution assay (SLDA)

A mammosphere formation assay was performed as previously described with the following modifications^60^. Briefly, ten thousand cells were plated on a six-well ultra-low attachment plate (Corning Inc.) and were grown in sphere formation medium, which is serum-free DMEM/F12 (1:1) medium supplemented with B27 (Invitrogen), 20 ng/ml epidermal growth factor, 1 μg/ml hydrocortisone, 5 μg/ml insulin, and 5 μg/ml β-mercaptoethanol. One ml of medium was added every other day for eight days. Images of mammospheres were recorded and the number of mammospheres was manually counted at day 5 and Day 8. Experiments were performed in duplicate and repeated two times. The sphere limiting dilution assay (SLDA) was performed as published paper^61^. Briefly, different titers (1000, 100, 50 and 1) of cells either control or treated with different drugs were plated in 96-well ultra-low attachment plate (Corning Inc.) in defined sphere formation medium as described above. Wells forming spheres after 12–14 days were scored as positive and data were analyzed using the ELDA software.

### Immunohistochemistry analysis

For H&E staining, paraffin-embedded sample slides were de-paraffinized, hydrated and then stained with hematoxylin for one minute. After rinsing, the slides were stained with eosin for one minute, rinsed and sealed with cover slips using Permount. For IHC, rabbit monoclonal anti-ALDH1A1 antibody (1:50 dilution, Abcam, #ab52492), rabbit polyclonal anti-CD44 antibody (pre-diluted Ab, IHC world, #IW-PA1021), rabbit polyclonal anti-cleaved caspase 3 antibody (1:50, Cell Signaling Technology), and rabbit polyclonal anti beta-tubulin (1:100 dilution, Abcam, #ab15568). These antibodies were used in conjunction with secondary kit, Pollink-2 plus HRP Rabbit with DAB, GBI labs, #D39-18.

### Drug testing and therapeutic evaluation *in vivo*

All animal studies were conducted with the approval and oversight of the institution’s veterinary staff and IACUC. All methods were performed in accordance with the relevant guidelines and regulations. A pilot study was performed to defined toxicity and dose ranges of DSF and BKM120. Eight week old NCR SCID female mice were implanted on day 0 bilaterally subcutaneously (S.C.) with MDA-MB468 tumor fragments via trocar. On day 3 (early stage disease), mice were randomly distributed to control and treatment groups (n = 2 mice/group), and treatment was started: Over a total treatment duration of 15 days, Disulfiram (Sigma, T1132) was given on a Q2d schedule (every other day) for a total of 344 mg/kg S.C., and BKM120 (Karebay Biochem Inc) was given daily at two dose levels (804 mg/kg and 515 mg/kg respectively), by oral gavage (P.O. = per os). For the combination groups, doses were matched to the single arm(s) of each drug. The experiment was terminated and the mice were euthanized when the tumor in mice reach 1500 mm^3^ or 2 mg. Tumor size was measured with calipers every other day. Tumor volume (V) was determined by the equation V = (L × W^2^) × 0.5, where L is the length and W is the width of the tumor.

For an *in vivo* evaluation of Taxol, BKM120 and Disulfiram in combination against the TNBC tumor xenograft model MDA-MB468, tumor fragments were implanted subcutaneously via trochar into 36 Female NCR-SCID mice on day 0. Mice were then randomly assigned into six groups (n = 6 mice/group) and treatment initiated on day 3 post implant (early stage disease): 1) Diluent Control; 2) Taxol 7.5 mg/kg BID IV q3dx6; total: 90 mg/kg; 3) Disulfiram (DSF) + BKM120 (BK): [DSF at 50 mg/kg SC q2dx9 (450 mg/kg total) and BK at 50 mg/kg PO q2dx9 (450 mg/kg total); 4) DSF + BK [DSF at 40 mg/kg SC both @ q2dx9 (360 mg/kg total) and BK at 32 mg/kg PO q2dx9 (288 mg/kg total)]; 5) DSF + BK + Taxol (DSF + BK dose match to Group 3 and Taxol dose match to Group 2; and 6) DSF + BK + Taxol (DSF + BK dose match to Group 4 and Taxol dose match to Group 2). The overall experimental endpoint was tumor burden (max = 2.0 g per mouse).

### Statistical Analysis

All data are presented as mean ± SEM except stated. For Figs 2–4,6B and 6D, as well as Supplementary Figs 1,3 and 4, one-way ANOVA with multiple comparisons was performed to compare the difference between each treatment group with the related control group. For the dose-outcome plots in Fig. 6, two-way ANOVA followed by multiple comparisons was used to compare the two values at a given concentration. Two-sample t-tests were performed to compare the apoptosis percentage between ALDH^+^ and ALDH^−^ cells or CD44^+^ and CD44^−^ cells in Fig. 5. For all analysis, *P* value less than 0.05 was considered statistically significant (*P < 0.05, **P < 0.01, ***P < 0.001, ****P < 0.0001).

Tumor growth curves with mean ± standard error were plotted and growth rates were tested with linear mixed model. Tumor latency to 1 g total burden was plotted and tested with Kruskal-Wallis test. Time to euthanasian were plotted with Kaplan-Meier method and compared with log-rank test. Tumor recurrence, a subgroup analysis among those mice whose tumors shrunk to below palpable size, was defined as days from day 0 to the day when tumor returns and reached 50 mg. Differences in tumor recurrence were tested with Kruskal-Wallis test. Analyses were performed using R version 3.3 (The R Foundation for Statistical Computing). A p-value of <0.05 was considered statistically significant.

## Electronic supplementary material

Supplementary information
