# Supplementary material for: Disulfiram and BKM120 in Combination with Chemotherapy Impede Tumor Progression and Delay Tumor Recurrence in Tumor Initiating Cell-Rich TNBC
Source: Sci Rep. 2019 Jan 18;9:236. doi: 10.1038/s41598-018-35619-6 (PMC6338744; doi:10.1038/s41598-018-35619-6)

Supplementary Information

Disulfiram and BKM120 in Combination with Chemotherapy Impede Tumor Progression and Delay Tumor Recurrence in Tumor Initiating Cell-Rich TNBC

Ling Wu[1], Fanyan Meng[2], Lun Dong[1,3], C. James Block[1], Allison V. Mitchell[1], Jason Wu[4], Hyejeong Jang[1], Wei Chen[1], Lisa Polin[1], Qifeng Yang[3], Q. Ping Dou[1], Guojun Wu[1, #]

Affiliation:

1. Barbara Ann Karmanos Cancer Institute, Department of Oncology, Wayne State University School of Medicine, 4100 John R, Detroit, MI, USA
2. Comprehensive Cancer Centre of Drum Tower Hospital, Medical School of Nanjing University and Clinical Cancer Institute of Nanjing University, Zhongshan Road, Nanjing, Jiangsu Province, P. R. China
3. Department of Breast Surgery, Qilu Hospital, Shandong University, Jinan, Shandong Province, P. R. China
4. Department of Biology, Purdue University, West Lafayette, IN, USA

#Corresponding Author:

Guojun Wu, Ph.D.
Associate Professor
Molecular Therapeutics Program
Karmanos Cancer Institute
Departments of Oncology
Wayne State University School of Medicine
4100 John R
HWCRC, Room 824
Detroit, MI 48201
313-576-8349 (O)
Email: wugu@karmanos.org

Supplementary Figure Legends

Supplementary Fig. 1. $ALDH^+$ and $CD49f^+/CD24^+$ TIC populations display different oncologic properties. A. The relative percentage of $CD49f^+/CD24^+$ and $ALDH^+$ TIC populations, as well as the overlap between them in 4T1 cells. a = bipotent; b = $ALDH^+$; c = $CD49f^+/CD24^+$; and d = bulk cells. **B and C.** Comparison of the invasive (B) and tumorsphere formation (C) capabilities of different TIC populations and bulk cells. a = bipotent; b = $ALDH^+$; c = $CD49f^+/CD24^+$; and d = bulk cells. One-way ANOVA followed by multiple comparisons was used with $P<0.05$ as statistically significant. **D.** The morphological differences between $ALDH^+$ and $CD49f^+/CD24^+$ TICs. **E.** The self-renewal capabilities of different TIC populations were determined by repopulation assay.

Supplementary Fig. 2. Generation of a chemo-resistant SUM159 cell model and analysis of TIC populations. A. The Schematic approach of generation of chemo-resistant SUM159 cells. Different doses of Paclitaxel, 40 nM, 70 nM, 90 nM and 120 nM were used sequentially to treat SUM159 cells. The selection duration of each treatment was two weeks. The stable cells in each round were analyzed for the TIC population redistribution. **B.** FACS analysis of $ALDH^+$ population of stable PAC resistant SUM159 cells for each dose of Pac treatment. **C.** Summary of the FACS analysis of the parental SUM159 cells and different Pac resistant SUM159 cells. Significant increase of $ALDH^+$ population was found in Pac-resistant SUM159 cells.

Supplementary Fig. 3. The effect of DSF, Cu and DSF-Cu on TIC population in cancer cells. A, B. 4T1 cells were treated with DSF, Cu and DSF-Cu mixture and FACS analysis was performed for $ALDH^+$ (**A**) and $CD49f^+/CD24^+$ (**B**) populations. DSF-Cu mixture, but not DSF or Cu individually, demonstrated significant effect on inhibiting $ALDH^+$ population. **C, D.** SUM102 cells were treated with DSF, Cu and DSF-Cu mixture and FACS analysis was performed for $ALDH^+$ (**C**) and $CD44^+/CD24^-$ (**D**) populations. Similar to above, DSF-Cu mixture, but not DSF or Cu individually, demonstrated significant effect on inhibiting $ALDH^+$ population. One-way ANOVA followed by multiple comparisons was used with $P<0.05$ as statistically significant.

Supplementary Fig. 4. Dose dependent effect of DSF-Cu on TIC populations in SUM102 cells. A. Human breast cancer SUM102 cells were treated with different doses of DSF-Cu mixture. $ALDH^+$ (top panels), but not the $CD44^+/CD24^-$ (low panels) TIC population were significantly decreased along with the increase of DSF-Cu mixture doses. **B.** Summary of the results of panel A. DSF-Cu mixture inhibit $ALDH^+$ population in a dose

dependent manner, while induce an minor increase of $CD44^+/CD24^-$ population. One-way ANOVA followed by multiple comparisons was used with $P<0.05$ as statistically significant.

Supplementary Fig. 5. The apoptosis inducing effect of DSF/Cu and BKM120 on TIC and non-TIC populations in breast cancer cells. A. Immunofluorescence analysis showed positive staining of the pro-apoptosis protein p27 (Red) and $ALDH^+$ (green) in SUM102 cells treated with DSF/Cu. White arrow points to strong staining cells and blue arrow points to weak staining. A correlation of p27 and ALDH staining was observed. **B.** Immunofluorescence analysis showed positive staining of the pro-apoptosis protein p27 (Red) and $CD44^+/CD24^-$ populations (green) in MDA-MB231 cells treated with BKM120. White arrow points to strong staining cells and blue arrow points to weak staining. A correlation of p27 and CD44 staining was observed.

Supplementary Fig. 6. Immunohistochemistry analysis of xenograft tumors. Tumors were collected from the MDA-MB468 tumor implanted NCR nu/nu mice with different treatments, as indicated at the top of the panels. The prepared tissue slides were analyzed with IHC using anti-ALDH1A1 (1:50 dilution, Abcam, #ab52492), CD44 (pre-diluted, IHC world, #IW-PA1021), Cleaved Caspase 3 (1:50, Cell Signaling Technology, #9664) and β-tubulin antibodies (1:100 dilution, Abcam, #ab15568), as well as H&E staining. Scale bars, 20 μm.

Supplementary Table 1. A pilot *in vivo* study to determine the dose range of BKM120 and DSF

Cage	Treatment	Drug Route	Schedule	Total Dosage mg/kg	Median Tumor Burden in mg on day 19 (Range)	% T/C
1	Diluent Control	PO & SC	qd 3-17 & q2d x 8 start d3	NA NA	851	–
2	Disulfiram	SC	q2d x 8 start d3	344	576	67
3	BKM120	PO	qd 3-17	804	231	27
4	BKM120	PO	qd 3-17	515	296	35
5	BKM120 + Disulfiram	PO & SC	qd 3-17 & q2d x 8 start d3	804 & 344	189 (one mouse died)	22
6	BKM120 + Disulfiram	PO & SC	qd 3-17 & q2d x 8 start d3	515 & 344	361	42

Supplementary Table 2. The *in vivo* investigation of BKM120 and DSF in combination with chemotherapy.

Cage	Treatment	Drug Route	Schedule	Total Dosage mg/kg	Mean Body Wt. Loss in g/mouse
1	Diluent Control	PO & SC & IV	Q2dx9 & Q2dx9 & BID q3dx6 Start d3	NA NA NA	+0.2
2	Taxol	IV	BID q3dx6 Start d3	90	-0.3
3	Disulfiram & BKM120	SC & PO	Q2dx9 & Q2dx9 Start d3	450 & 450	+1.3
4	Disulfiram & BKM120	SC & PO	Q2dx9 & Q2dx9 Start d3	360 & 288	+2.0
5	Taxol & Disulfiram & BKM120	IV & SC & PO	BID q3dx6 & Q2dx9 & Q2dx9 Start d3	90 & 450 & 450	-0.7
6	Taxol & Disulfiram & BKM120	IV & SC & PO	BID q3dx6 & Q2dx9 & Q2dx9 Start d3	90 & 360 & 288	+1.1

Supplementary Fig. 1, Wu et al.

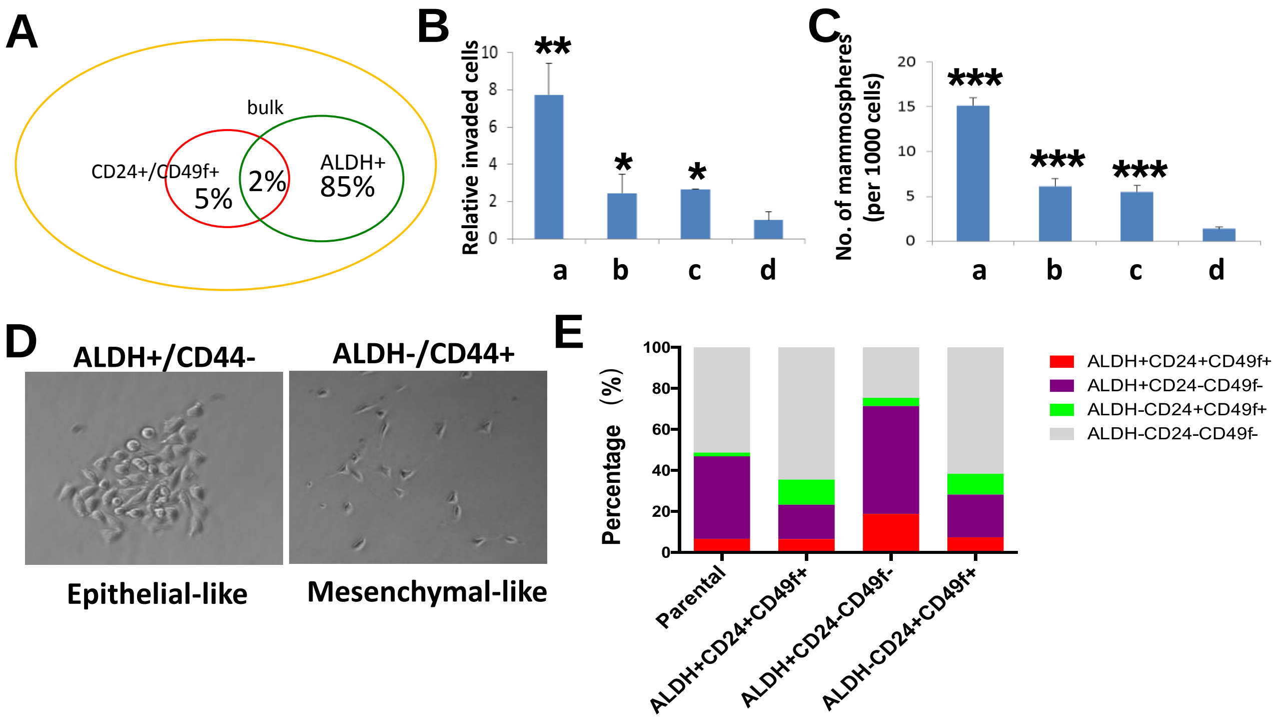

Supplementary Fig. 2, Wu et al.

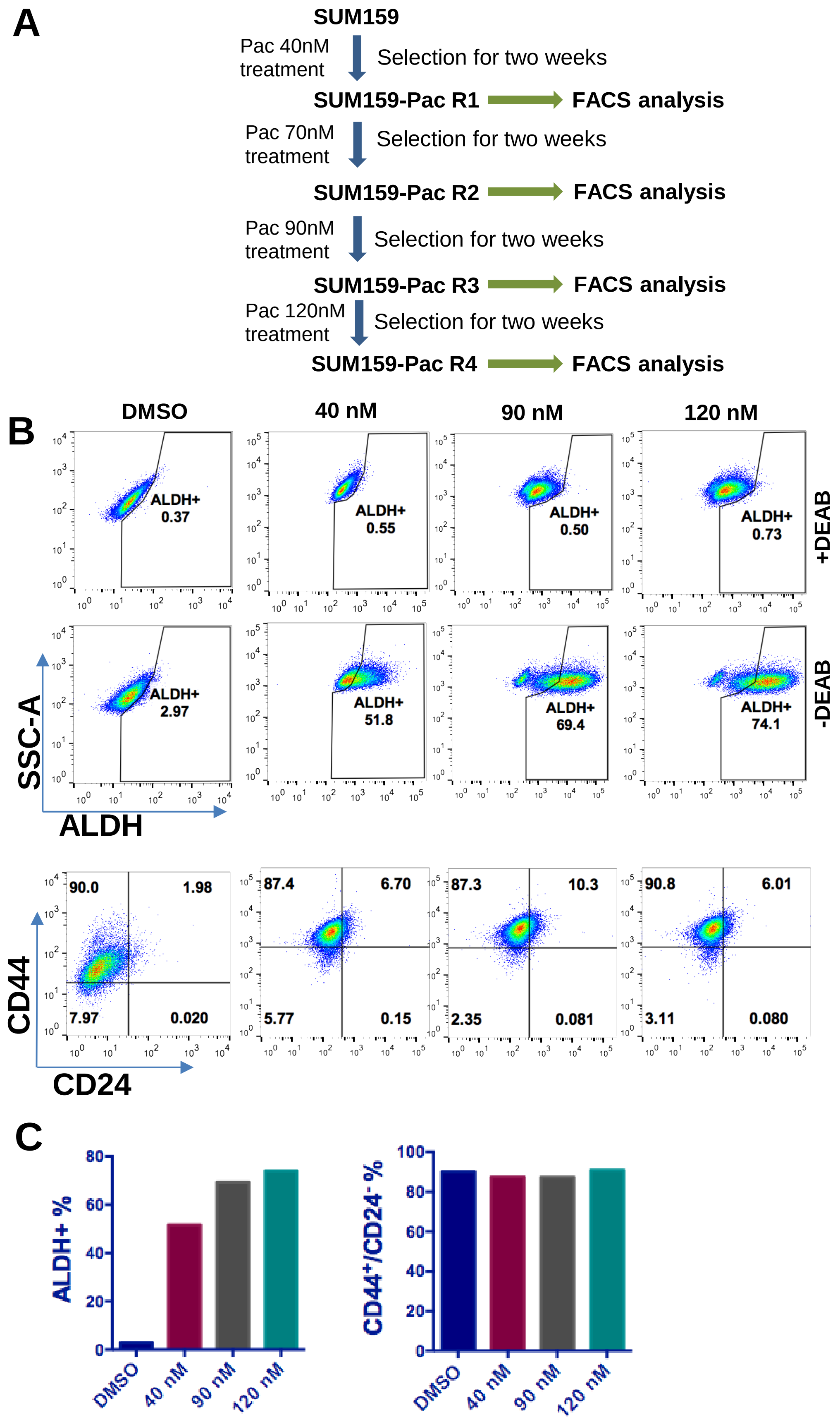

Supplementary Fig. 3, Wu et al.

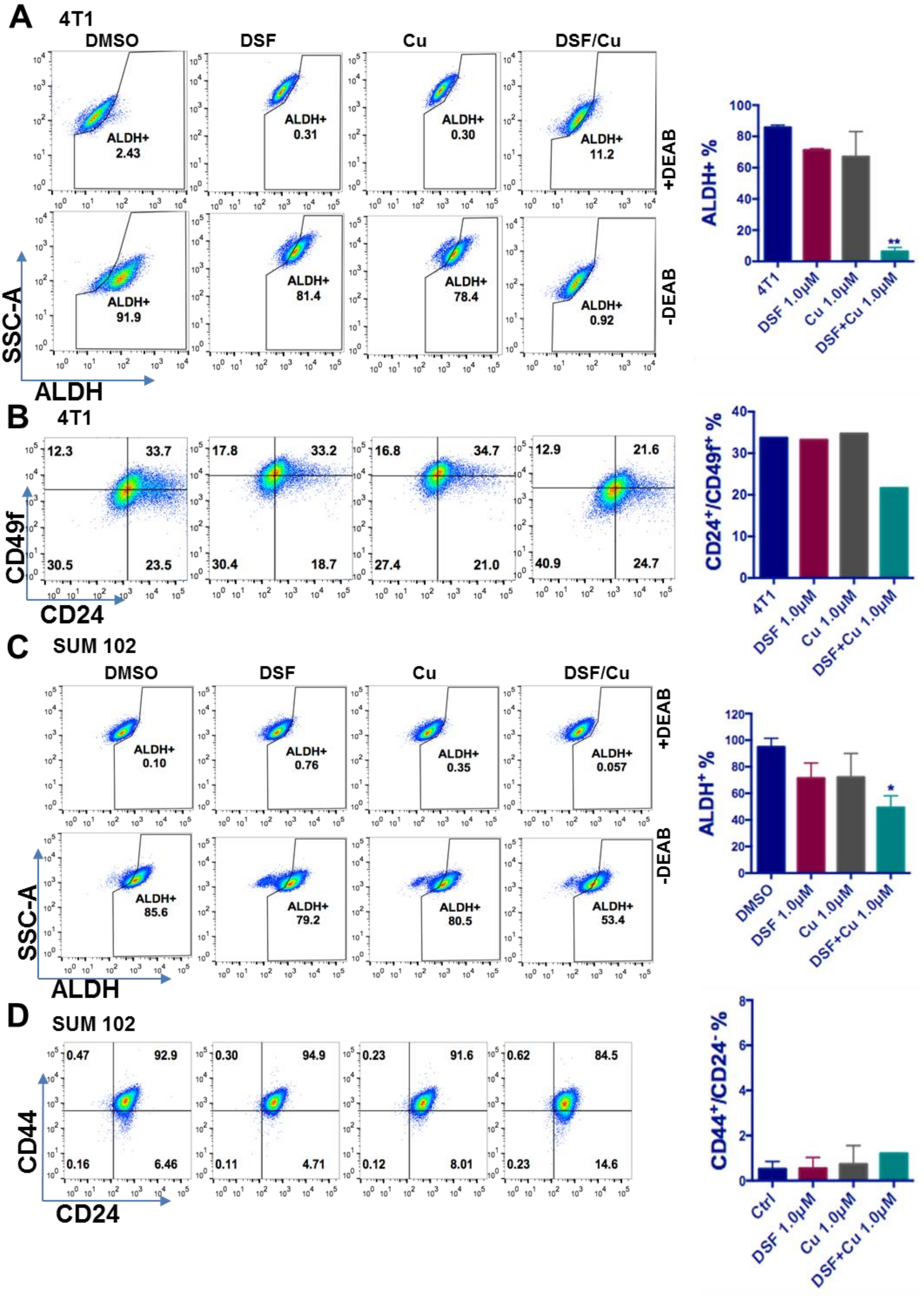

Supplementary Fig. 4, Wu et al.

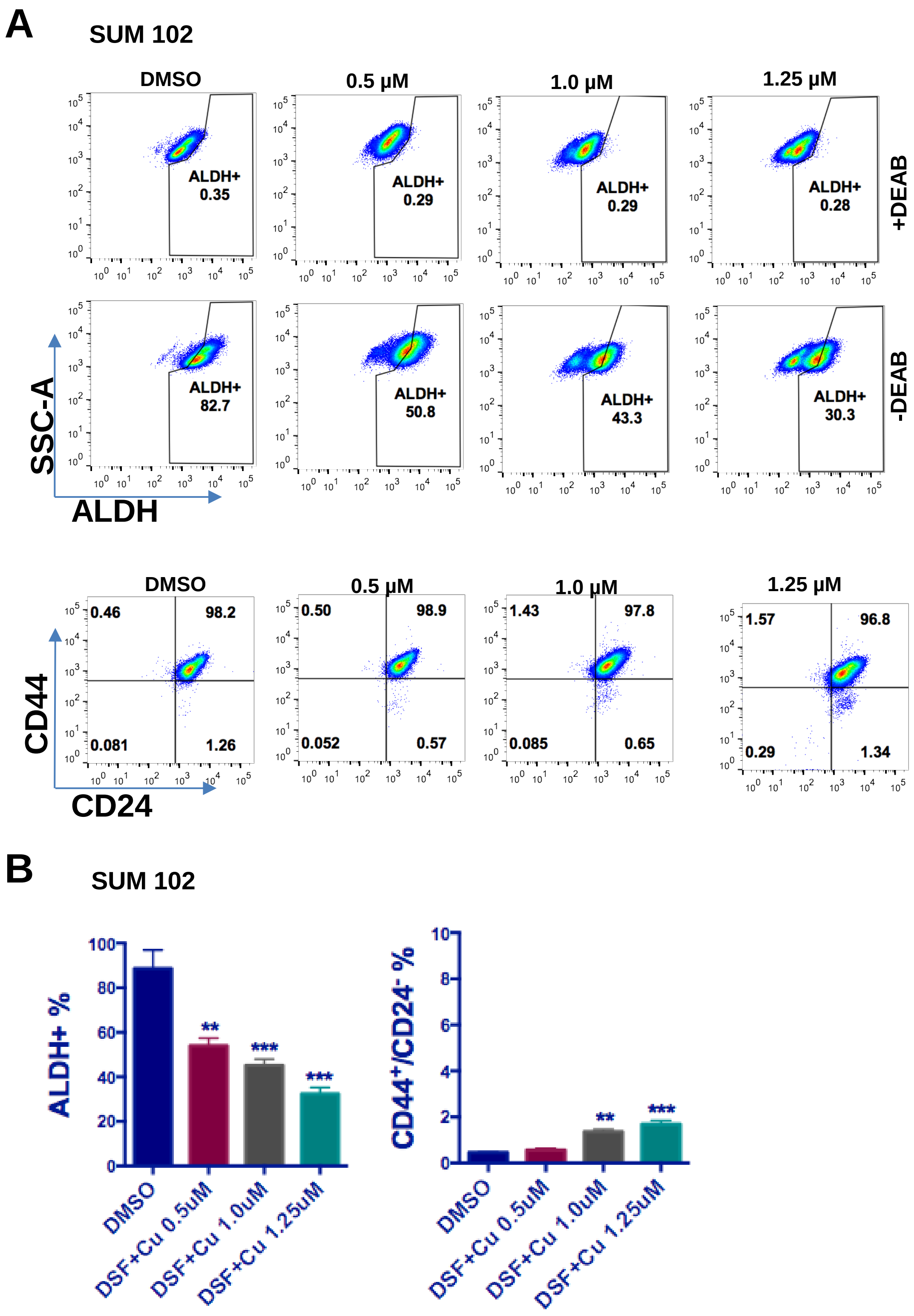

Supplementary Fig. 5, Wu et al.

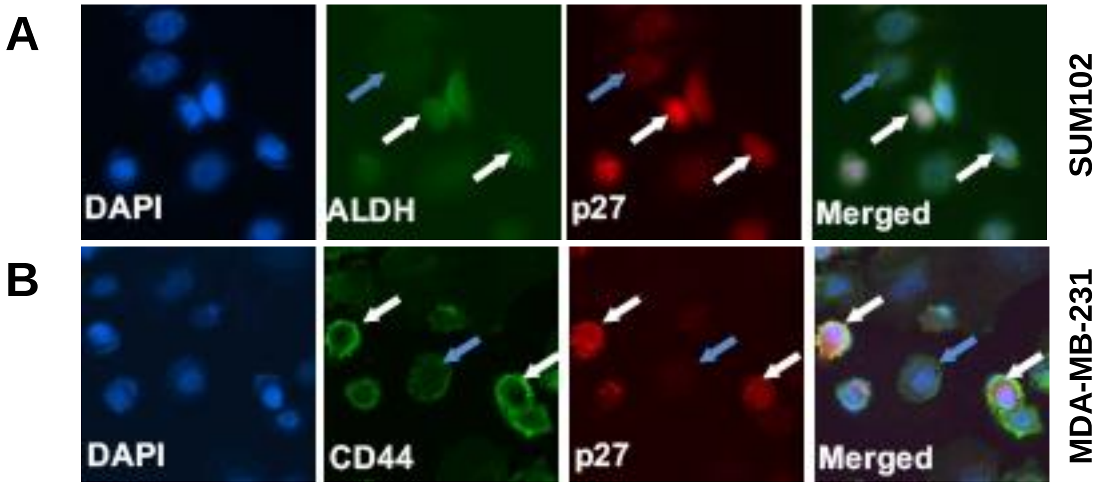

Supplementary Fig. 6, Wu et al.

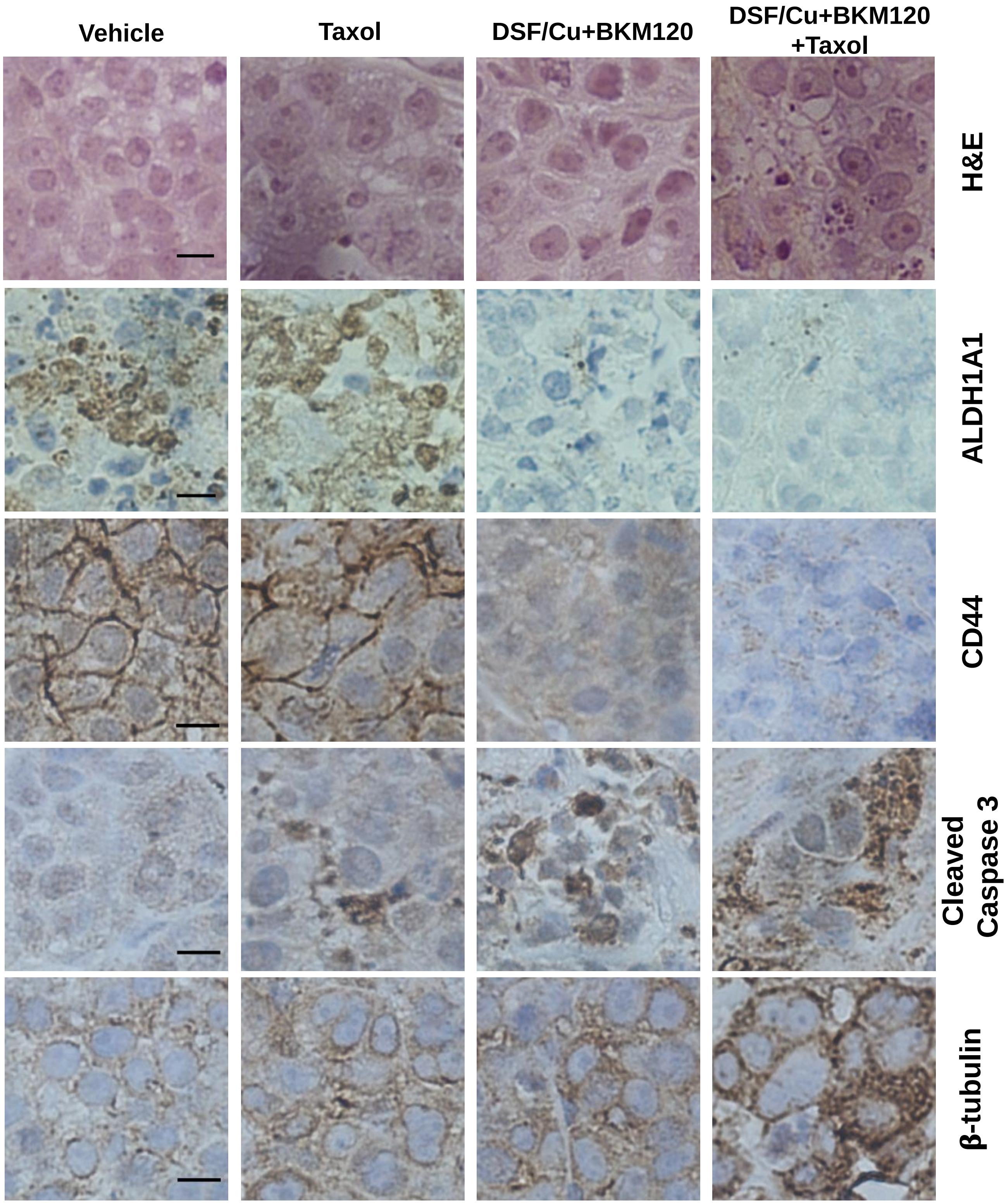

Supplement: Supplementary file 1 — Supplementary information [file 41598_2018_35619_MOESM1_ESM.pdf]
